# Differential Impacts of Soybean and Fish Oils on Hepatocyte Lipid Droplet Accumulation and Endoplasmic Reticulum Stress in Primary Rabbit Hepatocytes

**DOI:** 10.1155/2016/9717014

**Published:** 2016-01-05

**Authors:** Xueping Zhu, Zhihui Xiao, Yumin Xu, Xingli Zhao, Ping Cheng, Ningxun Cui, Mingling Cui, Jie Li, Xiaoli Zhu

**Affiliations:** ^1^Department of Neonatology, Children's Hospital of Soochow University, Suzhou, Jiangsu 215003, China; ^2^Department of Intervention, The First Affiliated Hospital of Soochow University, Suzhou 215006, China

## Abstract

Parenteral nutrition-associated liver disease (PNALD) is a severe ailment associated with long-term parenteral nutrition. Soybean oil-based lipid emulsions (SOLE) are thought to promote PNALD development, whereas fish oil-based lipid emulsions (FOLE) are thought to protect against PNALD. This study aimed to investigate the effects of SOLE and FOLE on primary rabbit hepatocytes. The results reveal that SOLE caused significant endoplasmic reticulum (ER) and mitochondrial damage, ultimately resulting in lipid droplets accumulation and ER stress. While these deleterious events induce hepatocyte injury, FOLE at high doses cause only minor ER and mitochondrial damage, which has no effect on hepatic function. SOLE also significantly upregulated glucose-regulated protein 94 mRNA and protein expression. These data indicate that SOLE, but not FOLE, damage the ER and mitochondria, resulting in lipid droplets accumulation and ER stress and, finally, hepatocyte injury. This likely contributes to the differential impacts of SOLE and FOLE on PNALD development and progression.

## 1. Introduction

Parenteral nutrition (PN) is a life-saving treatment for infants and children who are unable to adequately absorb crucial nutrients due to intestinal failure [1–3]. PN's wide pediatric application significantly improves patients' nutritional statuses, which avoids the necessity for complex gastrointestinal surgery, thus increasing patient survival rates [1–3]. Nevertheless, long-term PN administration can have severe deleterious health effects, such as promoting the development of life-threatening PN-associated liver disease (PNALD) [2]. PNALD is characterized by significant alterations to liver histology that range from hepatocyte steatosis and cholestasis to liver fibrosis, cirrhosis, or complete liver failure [2]. Importantly, PNALD is a major cause of morbidity and mortality in infants requiring long-term PN, with ten deaths and one referral for liver transplantation in the first year of life among 66 PN-associated cholestasis patients (17%) [4]. Of the 21 infants with a maximum conjugated bilirubin ≥10.0 mg/dL, death or liver transplantation occurred in eight (38%) [4].

The underlying cause of PNALD onset still remains unclear. However, there is increasing evidence suggesting that the vegetable oil-based lipid emulsions used in PN treatments, which are rich in *ω*-6 polyunsaturated fatty acids (PUFAs), likely promote PNALD development [2]. *ω*-6 PUFAs have been reported to increase oxidative stress, enhance inflammatory responses, and impede proper biliary secretion [5–7]. Fish oil is particularly rich in *ω*-3 PUFAs and has anti-inflammatory properties [8]. Studies in both humans and other mammals have revealed that fish oil and fish oil-based lipid emulsions (FOLE) can prevent and alleviate PNALD [9–14]. However, the underlying reason why vegetable oil and FOLE have such dramatically different effects on PNALD remains unclear.

Lipids droplets (LD) are highly dynamic organelles that are critical for lipid storage in nearly all cell types [15]. Both excessive and insufficient LD concentrations are associated with a variety of human diseases. Under stress conditions, such as excessive nutrient intake and drug-induced liver injury, hepatocyte LD accumulation is apparent. This LD accumulation is an early manifestation of liver injury and ultimately causes liver steatosis [16, 17].

The endoplasmic reticulum (ER) is a cellular organelle that functions in protein synthesis, folding, modification, assembly, transport, and storage. A lapse in ER function often results in the accumulation of misfolded proteins within the ER, ultimately causing ER stress and the activation of the unfolded protein response (UPS) [18, 19]. The glucose-regulated proteins (GRP), GRP78 and GRP94, play important roles during UPS to maintain ER homeostasis [20].

Several previous studies suggest that LD originates in the ER [17]. ER stress is thought to play a role in several liver diseases and to be related to lipid homeostasis [18]. In addition, the UPS is also activated in several liver diseases, including obesity-associated fatty liver disease, viral hepatitis, and alcohol-induced liver injury [18]. It was also recently reported that GRP94 is involved in liver tumorigenesis [21, 22]. Our previous studies also demonstrated that GRP94 mRNA and protein levels are upregulated in newborn rabbits that were intravenously treated with soybean oil-based PN. Nevertheless, GRP94 levels remained unchanged in newborn rabbits treated with fish oil-based PN [23]. We hypothesized that soybean oil-based lipid emulsions (SOLE) and FOLE may have distinctive effects on hepatocyte LD accumulation and ER stress. In this study, we use primary rabbit newborn hepatocytes to determine the effects of SOLE and FOLE on hepatocyte homeostasis.

## 2. Materials and Methods

### 2.1. Isolation and Treatment of Primary Hepatocytes

The animal protocols used in these studies were approved by the Animal Ethics Committee of the Children's Hospital of Soochow University. Eight seven-day-old New Zealand white rabbits (weighing 50–120 g) were obtained from Wuxi Huishan Jiangnan Experimental Animal Centre (animal license number SCXK [Su] 2009-0005) in Jiangsu, China. All rabbits were subjected to a 12/12-hour light-dark cycle and kept in an incubator that was maintained at 26°C to 28°C with 40% to 60% humidity.

Isolation of primary hepatocytes was performed using direct liver insolation and collagenase digestion, as previously reported [24]. Briefly, rabbits were anaesthetized with 3% pentobarbital sodium (1 mL/kg) before their livers were removed and placed in PBS at 4°C. Following several washes, livers were cut into multiple pieces (1 mm^3^). These pieces were centrifuged at 800 rpm/min for 6 min and the resulting pellet was collected and digested with collagenase I (Sigma-Aldrich, St. Louis, MO, USA) at 37°C for 30 min. The suspension was then filtered through a 100-mesh copper net. After centrifugation at 500 rpm/min for 10 min, hepatocytes were suspended in DMEM and centrifuged again. Following three cycles, hepatocytes were maintained in DMEM (Gibco of Thermo Fisher Scientific, Waltham, MA, USA) with 10% FBS (Sijiqing, Hangzhou, China) containing 100 U/mL penicillin, 100 U/mL streptomycin, 30 g/L glutamine, 5 *μ*g/mL insulin, and 1 × 10^−6^ mol/L hydrocortisone at a density of 2 × l0^4^ cells/mL. Cells were cultured in a 5% CO_2_ incubator at 37°C. Media were changed after 24 h, and then every two days thereafter. Primary hepatocytes were identified using periodic acid-Schiff staining (PAS). Cells were passaged upon reaching 80% confluence.

Primary hepatocytes were treated with varying concentrations of SOLE (0.2%, 0.4%, 1%, and 2% diluted in DMEM from 20% SOLE). The 0.4% and 1% concentrations were used for all follow-up experiments. Hepatocytes were then divided into five groups: control group (CON), 0.4% or 1% FOLE (diluted from 10% FOLE) treatment (FO Low, FO High) and 0.4% or 1% SOLE treatment (SO Low, SO High). SOLE (20%) and FOLE (10%) were both purchased from Sino-Swed Pharmaceutical, China.

### 2.2. MTT Assay

Primary hepatocytes were seeded into 96-well plates at a density of 2 × 10^4^ cells per well and treated as previously described. Cells were cultured for 24, 48, 72, or 96 h. At each indicated time, MTT was added to each individual well at a final concentration of 0.5 mg/mL. Cells were then incubated at 37°C for an additional 4 h. Following incubation, the supernatant was removed and the cells were lysed in 200 *μ*L DMSO (Sinopharm Chemical Reagent Co. Ltd., Shanghai, China). The absorbance of the blue formazan derivative was measured at 490 nm using a microplate reader (DNM-9602; Shengke, Shanghai, China). All measurements were performed in triplicate and all experiments were repeated three times.

### 2.3. LD Accumulation Detection Using Oil Red O Staining

Primary hepatocytes were seeded into 24-well plates with polylysine-pretreated slides at a density of 2 × 10^4^ cells per well. After 24 h, cells were treated with different lipid emulsion types and concentrations, as previously described. At 24, 48, 72, and 96 h posttreatment, the slides were fixed in 10% (v/v) formaldehyde for 10 min and then stained with red oil O solution (0.5% in isopropanol, w/v) for 15 min. Following three washes with distilled water, slides were stained with hematoxylin for 10 min. The stained slides were imaged using a light microscope (B-Type; Qiyue, Shanghai, China).

### 2.4. Biochemical Tests

Culture media from the five experimental groups were collected at 0, 24, 48, and 72 h. Various biochemical parameters, including total bilirubin (TBIL), direct bilirubin (DBIL), alanine aminotransferase (ALT), aspartate aminotransferase (AST), gamma glutamyltranspeptidase (*γ*-GT), total protein (TP), prealbumin (PA), albumin (ALB), triglycerides (TG), and lactate dehydrogenase (LDH), were measured from each sample using an automatic biochemical analyzer (LXTM20; Beckman Coulter, Inc., Brea, CA, USA).

### 2.5. Histologic Analysis Using Transmission Electron Microscopy (TEM)

Primary hepatocytes from all five groups were collected at 24 h posttreatment and centrifuged at 1500 rpm/min for 5 min. Cell pellets were fixed in 2.5% (v/v) glutaraldehyde (pH 7.4–7.6) for 2 h. Cells were then pelleted by centrifugation, washed with PBS, and then fixed with 1% (w/v) osmium tetroxide (Sigma-Aldrich) for 2 h at room temperature. Following dehydration with graded series of ethanol (70–100%), cells were embedded in propylene oxide and Epon (Sigma-Aldrich) and solidified in 100% epoxy resin at 60°C for 48 h in preparation for generating 90 nm ultrathin sections. Tissue sections were stained with uranyl acetate (2%) and lead citrate (1%) and then imaged using TEM (Tecnai G2 F20 S-TWIN; FEI, Hillsboro, OR, USA).

### 2.6. Quantitative RT-PCR

Total RNA was isolated from hepatocytes from each of the five experimental groups at 24, 48, and 72 h posttreatment using Trizol (Invitrogen of Thermo Fisher Scientific) and then reverse-transcribed into cDNA using reverse transcriptase (M-MLV; Promega Inc., Madison, WI, USA), according to the manufacturers' protocol. cDNA samples were then amplified using Real-Time PCR (LightCycler 480 II; Roche, Basel, Switzerland). Conditions for PCR amplification were set to an initial 95°C for 10 min, which was then followed by 40 cycles of 95°C for 15 s, 55°C for 30 s, and 72°C for 35 s. The GPR94 primers used were forward 5′-GACCCTCCAGCAGCATAA-3′ and reverse 5′-AGAAGCCGCTCAACAAAT-3′. The *β*-actin (control) primers used were forward 5′-GGTCGGAGTGAACGGATTT-3′ and reverse 5′-CTCGCTCCTGGAAGATGG-3′. Quantitative PCR was performed in triplicate, and the relative mRNA expression levels were analyzed using the 2^−ΔΔCt^ method.

### 2.7. Immunohistochemistry

Primary hepatocytes were seeded into 96-well plates with polylysine-pretreated slides at a density of 2 × 10^4^ cells per well. After 24 h, cells were treated with different lipid emulsions, as described above. The slides were fixed in 95% ethanol for 20 min at room temperature. After washing with PBS three times, cells were incubated first in blocking buffer at 37°C (PBS-containing goat serum) for at least 30 min, and then overnight at 4°C in binding buffer (PBS containing 3% BSA and 0.3% Triton X-100) containing rabbit anti-GRP94 polyclonal antibody (Maixin, Fuzhou, China) at a 1 : 200 dilution. After three PBS washes, cells were incubated with binding buffer containing HRP-conjugated goat anti-rabbit IgG (Maixin) at a 1 : 100 dilution for 1 h at room temperature. GRP94 staining was imaged under a light microscope using DAB solution (Maixin). Slides were then stained with hematoxylin for 10 min. Stained samples were then reexamined using the light microscope. Three visual fields were randomly selected under 400x magnification and the mean protein signal intensity was quantified using image-pro-plus software (Olympus, Inc., Tokyo, Japan).

### 2.8. Statistical Analyses

All data are represented as the mean ± standard deviation and were analyzed using SPSS 17.0 software (SPSS Inc., Chicago, IL, USA). The differences between groups were analyzed using a one-way ANOVA with a post hoc Bonferroni test. A *p* value <0.05 was considered as statistically significant.

## 3. Results

### 3.1. Analysis of Cell Viability

Primary hepatocytes were isolated and identified using PAS staining (see Supplementary Figure 1 in Supplementary Material available online at http://dx.doi.org/10.1155/2016/9717014). To determine the optimal lipid emulsion concentration to be used in these experiments, we first determined the extent of SOLE-induced cytotoxicity on hepatocytes at various time points using an MTT assay. As displayed in Supplementary Figure 2A, 0.2% and 0.4% SOLE had no detectable cytotoxic effects at all time points, 1% SOLE showed minor cytotoxicity at only 96 h, and 2% SOLE showed significant cytotoxicity at every time point. Therefore, 0.4% and 1% lipid emulsion concentrations were used for all follow-up experiments.

The SOLE and FOLE cytotoxicities at 0.2% and 0.4% concentrations were also examined. These data revealed that only 1% SOLE caused minor cytotoxicity at 96 h and FOLE had no cytotoxic effects at either of these two concentrations (Supplementary Figure 2B).

### 3.2. High SOLE Concentration Affects Hepatocyte Function

**Figure 1:**
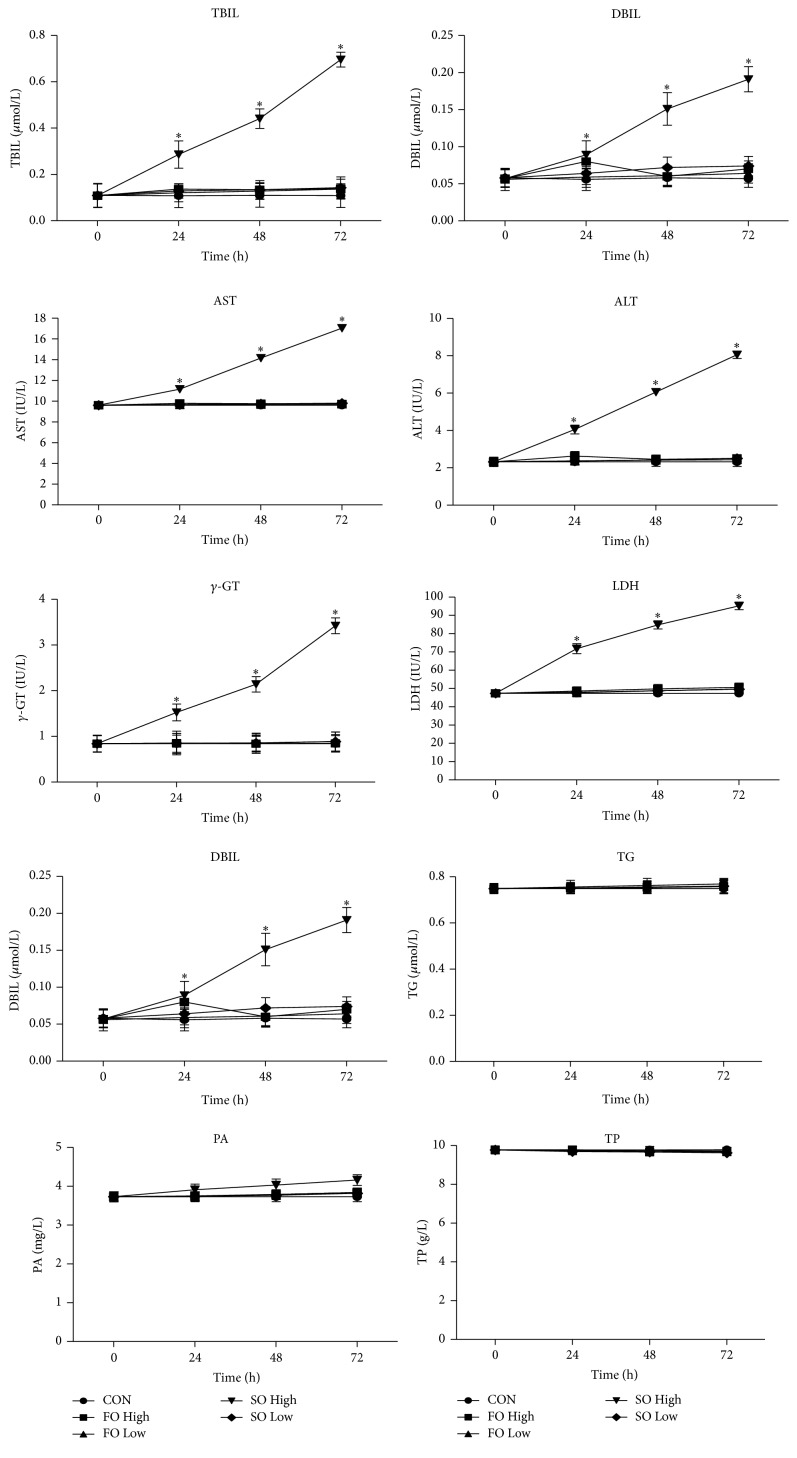
High concentrations of soybean oil affect hepatocyte function. Primary hepatocytes were treated with 0.4% or 1% soybean or fish oil-based lipid emulsions and several biochemical parameters were analyzed at the indicated time points to determine hepatic function; ^*∗*^ *p* < 0.05 versus CON. TBIL, total bilirubin; DBIL, direct bilirubin; ALT, alanine aminotransferase; AST, aspartate aminotransferase; *γ*-GT, gamma-glutamyltranspeptidase; TP, total protein; PA, prealbumin; ALB, albumin; TG, triglycerides; LDH, lactate dehydrogenase.

When PANLD occurs, several biochemical parameters including TBIL, DBIL, AST, and *γ*-GT are consistently increased. At the same time, liver damage destroys hepatocytes and alters their permeability, causing them to release more LDH and ALT. Liver damage also results in lower ALB synthesis. We therefore analyzed a variety of biochemical parameters to determine the effect of each treatment on hepatocyte function. As shown in Figure 1, the levels of TBIL, DBIL, ALT, AST, *γ*-GT, and LDH were all significantly higher in the SO High group when compared with all other groups at all time points (all *p* < 0.05). Furthermore, ALB levels were significantly lower in the SO High group compared with the other groups at each of the time points (all *p* < 0.05). No significant differences in TG, PA, or TP were observed among the groups at any of the time points. Additionally, no significant differences were observed in any of the parameters among the FO High, FO Low, SO Low, and CON groups at any of the time points.

### 3.3. Fish Oil and Soybean Oil Have Different Effects on Hepatocyte Function

**Figure 2:**
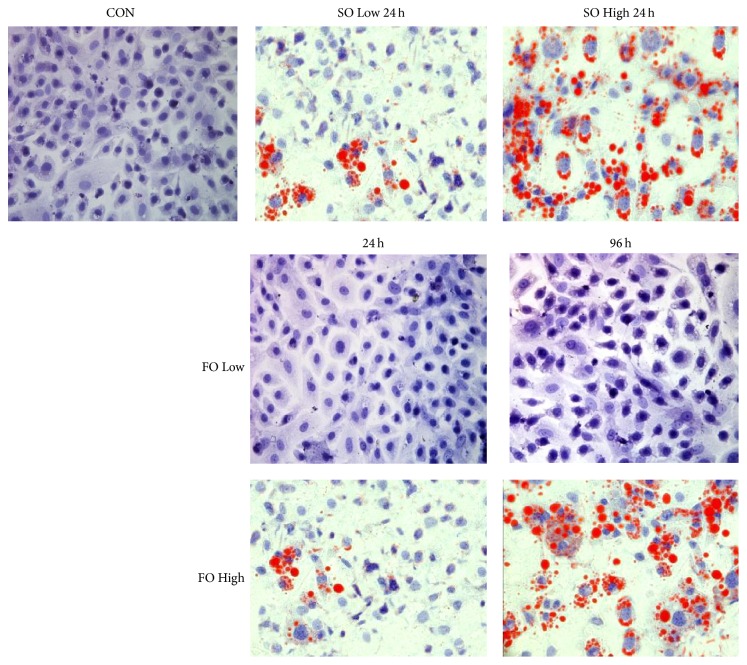
Soybean oils cause greater hepatocyte injury and LD accumulation than fish oil. Primary hepatocytes were treated with 0.4% or 1% soybean or fish oil-based lipid emulsions and cellular LD accumulation was detected using oil red O staining. LD, lipid droplets.

We analyzed the effects of FOLE and SOLE on hepatocyte LD accumulation using oil red O staining. As shown in Figure 2, hepatocytes in the CON group showed normal morphologies, with sharpened edges and limited red labeling at all time points. FO Low group hepatocytes showed normal morphologies at every time point and there were few red LD that were only visible at 96 h. The FO High group hepatocytes also exhibited normal morphologies at 24 and 48 h and showed minimal cell rounding at 72 h that compromised intercellular connections. In addition, red LD appeared in this group at 24 h and gradually increased throughout the subsequent time points. Nevertheless, although the SO Low group cells showed normal morphologies at all time points besides 96 h, including a few rounded cells, red LD appeared at 24 h and increased gradually in the proceeding time points. Moreover, cells in the SO High group became rounded at 24 h with many red LD also apparent, and these cells gradually exhibited blurred edges with LD fused circles around the inner membrane.

### 3.4. Soybean Oil Causes ER and Mitochondrial Damage

**Figure 3:**
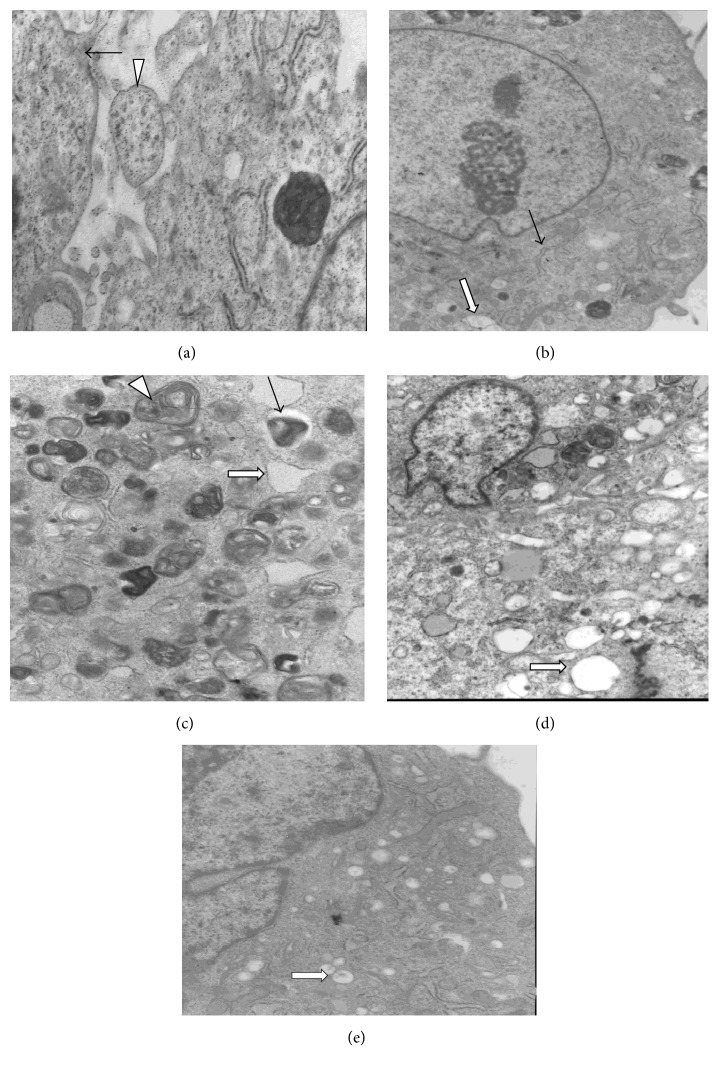
Soybean oils but not fish oils cause ER and mitochondrial damage. Primary hepatocytes were treated with 0.4% or 1% soybean (SO) or fish oil- (FO-) based lipid emulsions and the changes to both ER and mitochondrial morphologies were examined using TEM (×10000). (a) CON group, (b) FO High group, (c) FO Low group, (d) SO High group, and (e) SO Low group. White filled triangle indicates mitochondria; black filled arrow indicates ER; white filled arrow indicates lipid droplets. ER, endoplasmic reticulum; TEM, transmission electron microscopy.

We then used TEM to observe any alterations to hepatocyte ER morphology that were caused by the different lipid emulsions. Cells in the CON group demonstrated normal volumes, ER, and mitochondrial morphologies and lacked LDs (Figure 3(a)). Furthermore, cells in the FO High group showed slightly larger volumes, minor ER expansion, some mitochondrial swelling, the absence of mitochondrial crests, and few LDs (Figure 3(b)). Cells in the FO Low group exhibited normal cell volumes, normal ER and mitochondrial morphologies, and no LDs (Figure 3(c)). However, the majority of the cells in the SO High group showed a significantly larger volume, dramatic ER expansion, including a few fractured vacuole-containing ER, some mitochondrial swelling, the absence of mitochondria crests, and many LDs (Figure 3(d)). Similar to those in the FO High group, cells in the SO Low group also showed a slightly larger volume, minor ER expansion, minor mitochondrial swelling, the absence of mitochondria crests, and some LD (Figure 3(e)).

### 3.5. Soybean Oil Significantly Upregulates GRP94 mRNA and Protein Expression

**Figure 4:**
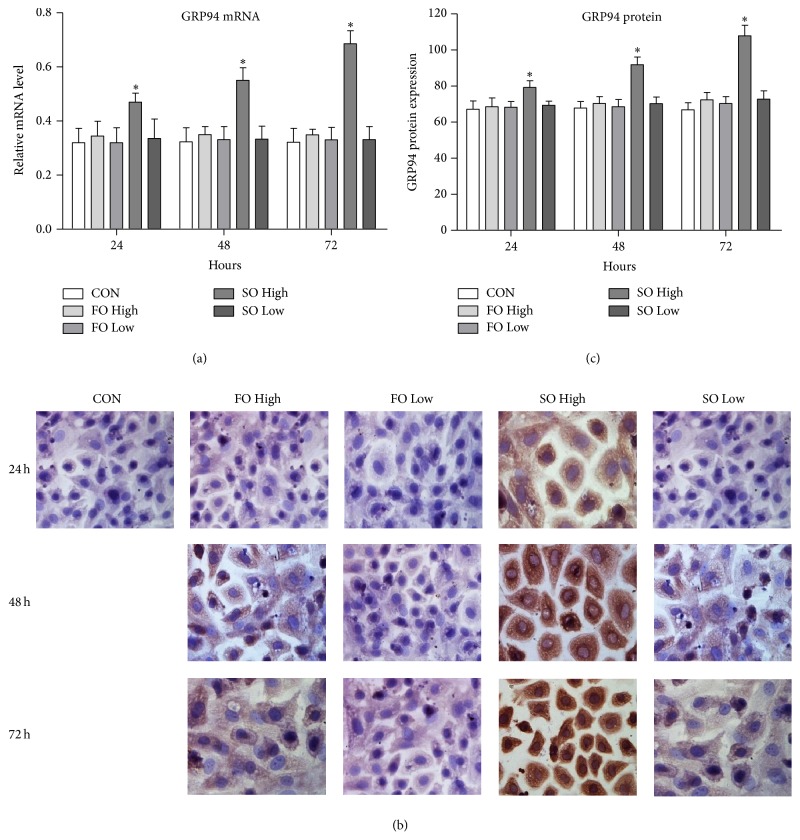
Soybean oils but not fish oils upregulate GRP94 mRNA and protein levels. Primary hepatocytes were treated with 0.4% or 1% soybean or fish oil-based lipid emulsions. The GPR94 mRNA expression (a) was detected using qRT-PCR and GRP94 protein expression (b, c) was detected by immunohistochemistry at the indicated time points; ^*∗*^ *p* < 0.05 versus CON. GRP, glucose-regulated protein.

We also determined the amount of GRP94 expression, which reflects the UPS during ER stress, in the different lipid emulsions. As shown in Figure 4, both the mRNA and protein levels of GRP94 in all except the SO high group showed no differences when compared with the CON group at any of the time points. The SO high group, however, showed significantly higher GRP94 expression levels at every time point (all *p* < 0.05).

## 4. Discussion

In this study, we reveal the differential effects of soybean and fish oil on hepatocyte function and structural integrity. The changes in several biochemical parameters that reflect hepatic function, which include LD accumulation, ER and mitochondrial structures, and ER stress, were compared between primary newborn rabbit hepatocytes that were treated with two different oil-based lipid emulsions. Our results reveal that SOLE caused significant damage to both the ER and mitochondria, ultimately resulting in LD accumulation, ER stress, and eventual hepatocyte malfunction. Conversely, FOLE caused only minor ER and mitochondrial damage at higher doses, which did not affect hepatic function. We therefore suggest that FOLE might serve a preventative and/or protective function against PNALD.

The lipid source in parenteral lipid emulsions is thought to play an essential role in the development of PNALD [9]. Currently, lipid emulsions are primarily derived from either soybean oils alone or a mixture of soybean and safflower oil. These vegetable oil-based lipid emulsions are rich in both phytosterols as well as *ω*-6 PUFAs. Phytosterols can accumulate in patient serum or in either cellular membranes or plasma lipoproteins, which are all associated with PNALD [25, 26]. Arachidonic acid is an *ω*-6 PUFA and is the precursor of the two important inflammatory mediators, namely, prostaglandins and leukotrienes. Therefore, *ω*-6 PUFAs may either initiate or exacerbate inflammation, as well as providing immunosuppressive effects, which could potentially contribute to PNALD development and progression [27, 28]. Contrarily, *ω*-3 PUFAs, which are abundant in fish oils, suppress inflammation by decreasing the production of inflammatory cytokines, eicosanoids, and reactive oxygen species [9, 29]. Our results also reveal that soybean oils cause significant ER and mitochondrial damage, ultimately resulting in LD accumulation within hepatocytes. Fish oil, on the other hand, showed no such effects. Hepatocyte injury can thus be alleviated in PN patients by using fish oils instead of vegetable oils in order to decrease the likelihood of causing PNALD.

Clinical trials suggest that the PNALD onset is positively correlated with both the timespan and dose of lipid emulsions applied [9, 30]. Our data are consistent with this correlation, revealing that increasing doses of soybean oil for longer timespans cause increasingly deleterious effects to hepatocyte function. High doses of fish oils over a long timespan also cause minor hepatocyte damage. However, although LD accumulation was consistently observed in SOLE-treated hepatocytes, there were no significant TG changes in the supernatant. The reason for this phenomenon could be attributable to the fact that the culture media used are different from the* in vivo* environment of hepatocytes. Whereas hormones in the body stimulate the release of TG, our culture media may not efficiently elicit this response.

Our results also show that while soybean oils induce ER stress and upregulate GRP94 expression in hepatocytes, fish oils have no such effect. ER stress is an adaptive cellular response to multiple stimuli and is linked to several diseases [31]. Persistent ER stress ultimately promotes apoptosis [19]. ER stress and the resulting apoptosis contribute to the development of many liver diseases, including alcoholic liver disease, nonalcoholic fatty liver disease, viral hepatitis, acute liver failure, and hepatocellular carcinoma [18, 32–34]. Previous work revealed that ER disorder and hepatic microvesicular steatosis were caused by the genetic ablation of ER stress-sensing pathways in mice [35]. In addition, the rescue of ER protein-processing capacity was shown to prevent the suppression of a subset of metabolic transcription factors that regulate lipid homeostasis. As hepatic steatosis also occurs during PNALD, it is possible that ER stress is also involved in this process.

GRP94 and GRP78 are two of the major proteins that are involved in UPS [36]. The upregulation of both GRP94 and GRP78 is a hallmark of ER stress. Our previous study demonstrated that GRP94 is upregulated in rabbits that receive PN containing soybean oil-based lipid emulsions. In this study, we further confirm that soybean oils directly affect hepatic function by upregulating GRP94. Recently, GRP94 was reported to be involved in hepatocellular carcinogenesis [37]. This suggests that soybean oil-driven GRP94 upregulation in hepatocytes may play an important role in PNALD development.

In conclusion, we reveal that soybean oils and not fish oils damage the ER and mitochondria, resulting in LD accumulation and ER stress. This ultimately induces hepatocyte injury, which likely explains the differential impacts of soybean and fish oils on PNALD development and progression.

## Supplementary Material

Answer: Isolation and identification of primary hepatocytes showed in Supplementary Figure 1A,B,C,D, and lipid emulsion cytotoxicity on hepatocytes using an MTT assay showed in Supplementary Figure 2A and B.
