# Supplementary figures and images for: Differential Impacts of Soybean and Fish Oils on Hepatocyte Lipid Droplet Accumulation and Endoplasmic Reticulum Stress in Primary Rabbit Hepatocytes
Source: Gastroenterol Res Pract. 2016 Jan 5;2016:9717014. doi: 10.1155/2016/9717014 (PMC4736330; doi:10.1155/2016/9717014)

Supplementary Figure 1.

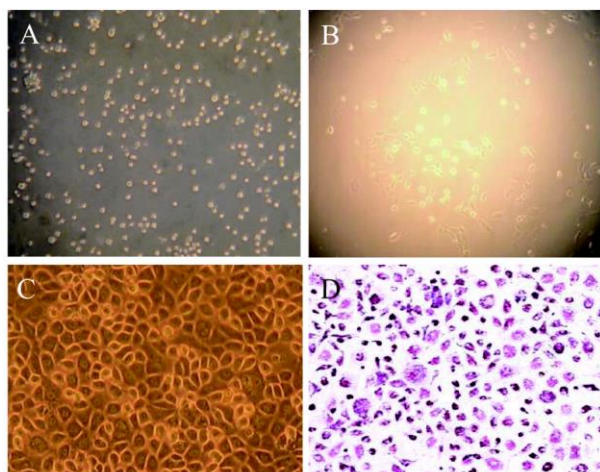

Supplementary Figure 2.

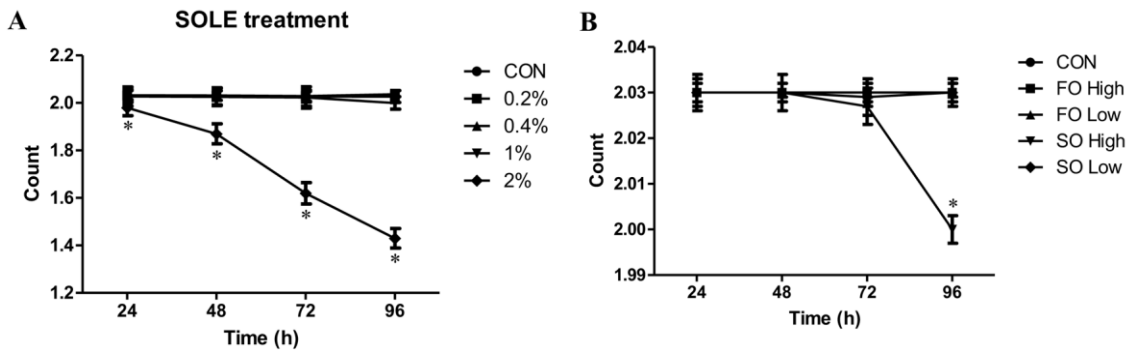

Supplement: Supplementary file 1 — Answer: Isolation and identification of primary hepatocytes showed in Supplementary Figure 1A,B,C,D, and lipid emulsion cytotoxicity on hepatocytes using an MTT assay showed in Supplementary Figure 2A and B. [file 9717014.f1.pdf]
